# Resting State Functional Connectivity of the Lateral and Medial Hypothalamus in Cocaine Dependence: An Exploratory Study

**DOI:** 10.3389/fpsyt.2018.00344

**Published:** 2018-07-27

**Authors:** Sheng Zhang, Wuyi Wang, Simon Zhornitsky, Chiang-shan R. Li

**Affiliations:** ^1^Department of Psychiatry, Yale University School of Medicine, New Haven, CT, United States; ^2^Department of Neuroscience, Yale University School of Medicine, New Haven, CT, United States; ^3^Interdepartmental Neuroscience Program, Yale University, New Haven, CT, United States; ^4^Beijing Huilongguan Hospital, Beijing, China

**Keywords:** resting state functional connectivity, hypothalamus, cocaine, craving, fMRI

## Abstract

The role of dopamine in cocaine misuse has been extensively documented for the mesocorticolimbic circuit. Preclinical work from earlier lesion studies to recent multidisciplinary investigations has suggested that the hypothalamus is critically involved in motivated behavior, with the lateral and medial hypothalamus each involved in waking/feeding and resting/satiety. However, little is known of hypothalamus function and dysfunction in cocaine misuse. Here, we examined resting state functional connectivity of the lateral and medial hypothalamus in 70 individuals with cocaine dependence (CD) and 70 age as well as gender matched healthy controls (HC). Image pre-processing and analyses followed published work. Compared to HC, CD showed increased lateral hypothalamic connectivity with dorsolateral prefrontal cortex and decreased functional connectivity with the ventral precuneus. CD showed increased medial hypothalamic connectivity with the inferior parietal lobule and decreased connectivity with the ventromedial prefrontal cortex, temporal gyrus, fusiform gyrus, and ventral striatum. Further, at trend level significance, the connectivity strength between lateral hypothalamus and dorsolateral prefrontal cortex was positively correlated with total amount of cocaine use in the past month (*p* = 0.004, *r* = 0.35) and the connectivity strength between medial hypothalamus and ventral striatum was negatively correlated with cocaine craving as assessed by the Tiffany Cocaine Craving Questionnaire (*p* = 0.008, *r* = −0.33). Together, the findings demonstrated altered resting state functional connectivity of the hypothalamus and may provide new insight on circuit level deficits in cocaine dependence.

## Introduction

Individuals with drug addiction are characterized by severe motivation deficits (1) and under-responsiveness to natural reinforcers (2–4). The dopaminergic pathways process reinforcing stimuli and play a critical role in motivated behavior (5–8). Dopamine helps not only to establish the motivational value of extrinsic stimuli during initial conditioning but also link incentives to action through learning (1, 6). Preclinical studies showed that repeated administration of stimulants altered dopaminergic signaling and motivated behaviors such as preference for sucrose (9–12). Abundant research of dopaminergic deficits in addiction has focused on the ventral striatum, largely in the context of reward-related responses. The hypothalamus also receives extensive projections from the dopaminergic midbrain and is implicated in functions from those essential to survival to cognitive and affective processes in support of goal-directed behavior (13–18). On the other hand, it remains unclear how hypothalamus function is influenced by cocaine misuse.

Located below the thalamus, the hypothalamus lies along third ventricle walls below the hypothalamic sulcus and continues across the floor of the ventricle (19, 20). The hypothalamus regulates arousal, food intake, sexual drive, reward and affective response (21–23). Previous studies have implicated the hypothalamus in drug addiction (24, 25). In rodents the transition from controlled to compulsive cocaine self-administration was associated with substantial remodeling of hypothalamic circuitry (26–28). Compulsive cocaine use altered an array of dopamine gene expression, leading to functional reorganization of the hypothalamus (26). In humans individuals with cocaine dependence (CD) showed altered hypothalamus activation viewing erotic vs. neutral pictures, as compared to healthy controls (HC) (29). Hypothalamus response to monetary reward vs. non-reward was associated with the duration of abstinence in CD (30). HC showed increased hypothalamus activation viewing food vs. neutral pictures (31–34), whereas a recent study reported decreased hypothalamus activation in CD for the same contrast (35). Together, these findings highlighted hypothalamus dysfunction in the context of reward, food and sex drive in cocaine addiction.

Preclinical work from earlier lesion studies to recent multidisciplinary investigations has suggested that the hypothalamus could be broadly divided into lateral hypothalamus (LH) and medial hypothalamus (MH) each involved in waking/feeding and resting/satiety (36). On the other hand, imaging studies in humans have not aimed to distinguish LH and MH and delivered a more diverse picture of hypothalamus functions. For instance, on the basis of reported coordinates, both LH (37–40) and MH (32–34) appeared to respond to exposure to high caloric vs. low caloric food or non-food stimuli. Both LH (41–49) and MH (46, 49–51) responded to exposure to erotic vs. neutral visual stimulation. Similarly, both LH (52–54) and MH (52, 55–57) increased activations to gain vs. no-gain scenarios in the monetary incentive delay task. Thus, both hypothalamus divisions appeared to be engaged in behavioral challenges that involved explicit reward.

Combining T1-weighted 3D Fast Field Echo MR imaging and histology, Baroncini and colleagues identified hypothalamic nuclei and adjacent white matter fascicles (20). Gray and white matter structures within and around the hypothalamus were identified via specific landmarks on histological sections, from the optic chiasm anteriorly to the mammillary bodies posteriorly, and with reference to published work and atlas (58–65). The landmarks, including the optic tract, the floor of the diencephalon, the third ventricle, and the fornix, were readily identifiable in MR scans. Identifiability scorings between an anatomist and a neuroradiologists for 20 brains was highly in agreement (Cohen's κ = 0.96, *p* < 0.0001). The averaged MNI coordinates of identified hypothalamic nuclei in anatomical MNI space were provided from the 20 volunteers. Based on these coordinates, the MH (mean: ±4, −2, −12) included the arcuate nucleus, ventromedial nucleus and part of the dorsomedial nucleus and LH (mean: ±6, −9, −10) included the other hypothalamic nuclei located laterally and posteriorly. With the coordinates, a more recent study highlighted distinct resting state functional connectivity (rsFC) of the LH and MH (66). Specifically, LH was more heavily connected to the dorsomedial PFC (dmPFC), thalamus, and frontal operculum, and MH was more connected to the vmPFC and ventral striatum. These studies suggested the feasibility in examining activity and functional connectivity of the LH and MH separately.

In the current study, we examined the rsFC of the LH and MH in CD as compared to HC. We hypothesized that (1) LH and MH would show distinct functional connectivities, in replication of Kullmann et al. (66); and (2) compared to HC, CD showed altered rsFC in correlation with clinical characteristics such as cocaine craving score and duration and recent amount of cocaine use. Further, the literature supports sex differences in hypothalamus dysfunction in relation to cocaine misuse. For instance, female as compared to male rats showed greater hypothalamic-pituitary-adrenal (HPA) axis activation following administration of cocaine (67). Male but not female rats showed significant increases in cocaine- and amphetamine-regulated transcript (CART) peptide expression in the hypothalamus following forced swim stress (68). In humans, cocaine use appeared to alter hypothalamic-pituitary-gonadal function more in men than in women (69). During exposure to stress or drug cues, cocaine-dependent women demonstrated more blunted HPA axis response than did cocaine-dependent men (70, 71). Thus, we explored potential sex differences in the current study.

## Materials and methods

This is an exploratory study using an imaging data set collected earlier (72). Briefly, cocaine dependent subjects (CD) were recruited for inpatient stay at the Clinical Neuroscience Research Unit of the Connecticut Mental Health Center and healthy control subjects (HC) participated in the study as “outpatients.” The goals of the original study were to examine the component neural processes of cognitive control using a stop signal task (73, 74) and how these processes were altered in cocaine addiction. The original sample consisted of 97 CD and 96 HC. The current study was based on a subsample of the participants who were also scanned during resting state. Below is a description of the subjects, study procedures, and data analysis specific to the current work.

### Subjects, informed consent, and assessment

Seventy recently abstinent subjects with cocaine dependence (CD, 52 men) and 70 age- and gender-matched healthy control (HC, 46 men) subjects participated in the study (Table 1). CD met criteria for current cocaine dependence, as diagnosed by the Structured Clinical Interview for DSM-IV (75). Recent cocaine use was confirmed by urine toxicology screens. They were drug-free while staying in an inpatient unit for 7–10 days prior to the current fMRI study. All subjects were physically healthy with no major medical illnesses or current use of prescription medications. None reported having a history of head injury or neurological illness. Other exclusion criteria included dependence on another psychoactive substance (except nicotine) and current or past history of psychotic disorders. Individuals with current depressive or anxiety symptoms requiring treatment or currently being treated for these symptoms were excluded as well. The Human Investigation committee at Yale University School of Medicine approved all study procedures, and all subjects signed an informed consent prior to participation.

**Table 1:** Demographics of the subjects.

**Subject characteristic**	**CD (*n* = 70)**	**HC (*n* = 70)**	***p*-value**
Age (years)	40.7 ± 7.7	38.5 ± 9.4	0.14^*^
Gender (M/F)	52/18	46/24	0.28^∧^
Current cigarette smokers/non-smokers	51/19	21/49	<0.001^∧^
Years of alcohol use	18 ± 9.0	19 ± 11.7	0.48^*^
Amount of average monthly cocaine use (gm) in the prior year	26.1 ± 32.0	N/A	N/A
Average cocaine amount per use (gm)	1.4 ± 1.3	N/A	N/A
Days of cocaine use in the prior month	15.9 ± 9.9	N/A	N/A
Years of cocaine use	19.2 ± 8.0	N/A	N/A

values are mean ± S.D.;

* two-tailed two-sample t-test;

∧ *χ^2^ test*.

CD's were assessed with the Beck Depression Inventory (76) and the State-Trait Anxiety Inventory (77) at admission. The mean (±SD) BDI (10.0 ± 7.5) and STAI state (34.0 ± 8.1) and trait (37.8 ± 8.2) scores were within the range reported previously for individuals with cocaine dependence (78–81). Cocaine craving was assessed with the Cocaine Craving Questionnaire, brief version (CCQ-Brief), for all participants every 2–3 days during the inpatient stay (82). The CCQ-Brief is a 10-item questionnaire, abbreviated from the CCQ-Now (83). CCQ-Brief, CCQ-Now and other measures were highly correlated in craving assessment (82). Each item was rated on a scale from 1 to 7, with a higher total score (ranging from 10 to 70) indicating greater craving. Here, CDs averaged 22.7 ± 11.9 in CCQ score across all assessments and 19.3 ± 6.6 on the day or within 2 days of the scan.

### Imaging protocol

Conventional T1-weighted spin echo sagittal anatomical images were acquired for slice localization using a 3T scanner (Siemens Trio). Anatomical images of the functional slice locations were next obtained with spin echo imaging in the axial plane parallel to the AC–PC line with *TR* = 300 ms, *TE* = 2.5 ms, bandwidth = 300 Hz/pixel, flip angle = 60°, field of view = 220 × 220 mm, matrix = 256 × 256, 32 slices with slice thickness = 4 mm and no gap. Functional, blood oxygen level-dependent (BOLD) signals were then acquired with a single-shot gradient echo echoplanar imaging (EPI) sequence. 32 axial slices parallel to the AC–PC line covering the whole brain were acquired with *TR* = 2,000 ms, *TE* = 25 ms, bandwidth = 2,004 Hz/pixel, flip angle = 85°, field of view = 220 × 220 mm, matrix = 64 × 64, 32 slices with slice thickness = 4 mm and no gap. One 10-min resting state BOLD scan was obtained for each participant with eyes closed.

### Imaging data preprocessing

Data were analyzed with Statistical Parametric Mapping (SPM8, Wellcome Department of Imaging Neuroscience, University College London, U.K.). Images from the first five TRs at the beginning of each trial were discarded to enable the signal to achieve steady-state equilibrium between RF pulsing and relaxation. Standard image preprocessing was performed. Images of each individual subject were first realigned (motion corrected) and corrected for slice timing. A mean functional image volume was constructed for each subject per run from the realigned image volumes. These mean images were co-registered with the high-resolution structural image and then segmented for normalization with affine registration followed by nonlinear transformation (84, 85). The normalization parameters determined for the structure volume were then applied to the corresponding functional image volumes for each subject. Finally, the images were smoothed with a Gaussian kernel of 4 mm at Full Width at Half Maximum.

Additional preprocessing was applied to reduce spurious BOLD variances that were unlikely to reflect neuronal activity (86–89). The sources of spurious variance were removed through linear regression by including the signal from the ventricular system, white matter, and whole brain, in addition to the six parameters obtained by rigid body head motion correction. First-order derivatives of the whole brain, ventricular and white matter signals were also included in the regression.

Cordes and colleagues suggested that BOLD fluctuations below a frequency of 0.1 Hz contribute to regionally specific BOLD correlations (90). Thus, we applied a temporal band-pass filter (0.009 Hz < *f* < 0.08 Hz) to the time course in order to obtain low-frequency fluctuations, as in previous studies (87–89, 91).

### Head motion

As extensively investigated in Van Dijk et al. (92), micro head motion (>0.1 mm) is an important source of spurious correlations in resting state functional connectivity analysis (92). Therefore, we applied a “scrubbing” method proposed by Power and colleagues (93) and successfully applied in previous studies (94, 95) to remove time points affected by head motions. Briefly, for every time point t, we computed the framewise displacement given by *FD*(*t*) = |Δ*d*_*x*_(*t*)| + |Δ*d*_*y*_(*t*)| + |Δ*d*_*z*_(*t*)| + |Δα(*t*)| + |Δβ(*t*)| + |Δγ(*t*)|, where (*d*_*x*_, *d*_*y*_, *d*_*z*_) and (α, β, γ) are the translational and rotational movements, respectively (93). The second head movement metric was the root mean square variance (DVARS) of the differences in % BOLD intensity I(t) between consecutive time points across brain voxels, computed as follows: $DVARS\left(t\right)\;=\;\sqrt{\langle {\left|I\left(t\right)\;-\;I\left(t\;-\;1\right)\right|}^{2}\rangle }$, where the brackets indicate the mean across brain voxels. Finally, to compute each subject's correlation map, we removed every time point that exceeded the head motion limit FD(t) >0.5 mm or DVARS(t) >0.5% (93, 95). On average, 1% of the time points were removed across subjects. CD and HC did not differ in FD (*p* = 0.72) or in DVARS (*p* = 0.52).

### Seed based correlation and group analyses

**Figure 1:**
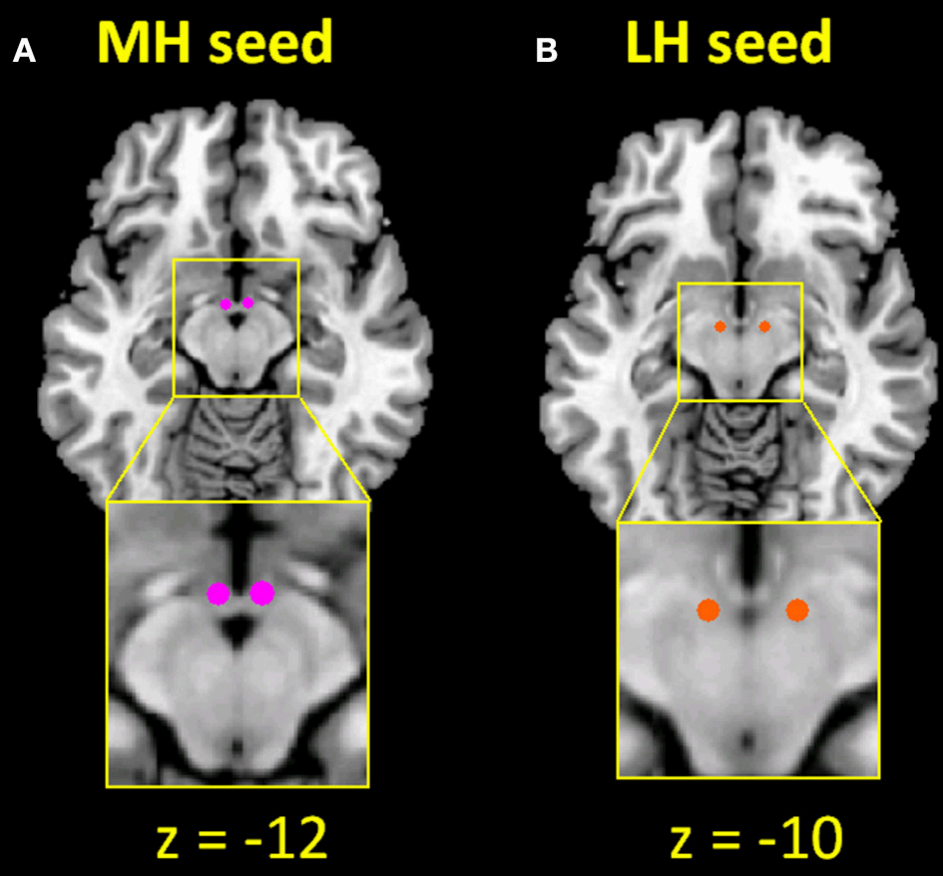
Seed regions of the **(A)** medial hypothalamus (MH; *x* = ±4, *y* = −2, *z* = −12) and **(B)** lateral hypothalamus (LH; *x* = ±6, *y* = −9, *z* = −10).

The seed regions of MH (two spheres of 2 mm in radius, centered at *x* = ±4, *y* = −2, *z* = −12) and LH (two spheres of 2 mm in radius, centered at *x* = ±6, *y* = −9, *z* = −10) were generated according to previous studies (Figure 1) (20, 66). Supplementary Figure 1 shows the seed region in relation to potential locations of the hypothalamic subnuclei based on an atlas (65). The BOLD time courses were averaged spatially over each of the MH and LH seeds. For individual subjects, we computed the correlation coefficient between the averaged time course of each seed region and the time courses of all other brain voxels. To assess and compare the resting state functional connectivity, we converted these image maps, which were not normally distributed, to z score maps by Fisher's z transform (96, 97): $z\;=\;0.5log_{e}\left[\frac{1\;+\;r}{1-r}\right]$. The Z maps were used in group random effect analyses. We performed one-sample *t*-test each on the Z maps of MH and LH for CD and HC and two-sample *t*-test with age as covariate to compare the two groups. We also performed a two-way ANOVA with age as a covariate on Z maps to verify group (CD vs. HC) main effect and examine seed (LH vs. MH) main effect and group by seed interaction. In addition, we examined main effect and interaction effect of sex differences in another two-way ANOVA (group × sex) each for LH and MH connectivity.

## Results

We reported the results of one-sample *t*-test of whole-brain connectivity of the MH and LH each for CD and HC and of two-sample *t*-test of CD vs. HC. The one-sample *t*-test allowed us to examine whether the findings of HC replicated those reported in earlier studies. Further, with a two-way ANOVA (group: CD vs. HC × seed: LH vs. MH) we examined whether LH and MH connectivities were differentially altered in CD vs. HC. All findings were queried at a corrected threshold, according to current reporting standards.

### Resting state functional connectivity (rsFC)

**Figure 2:**
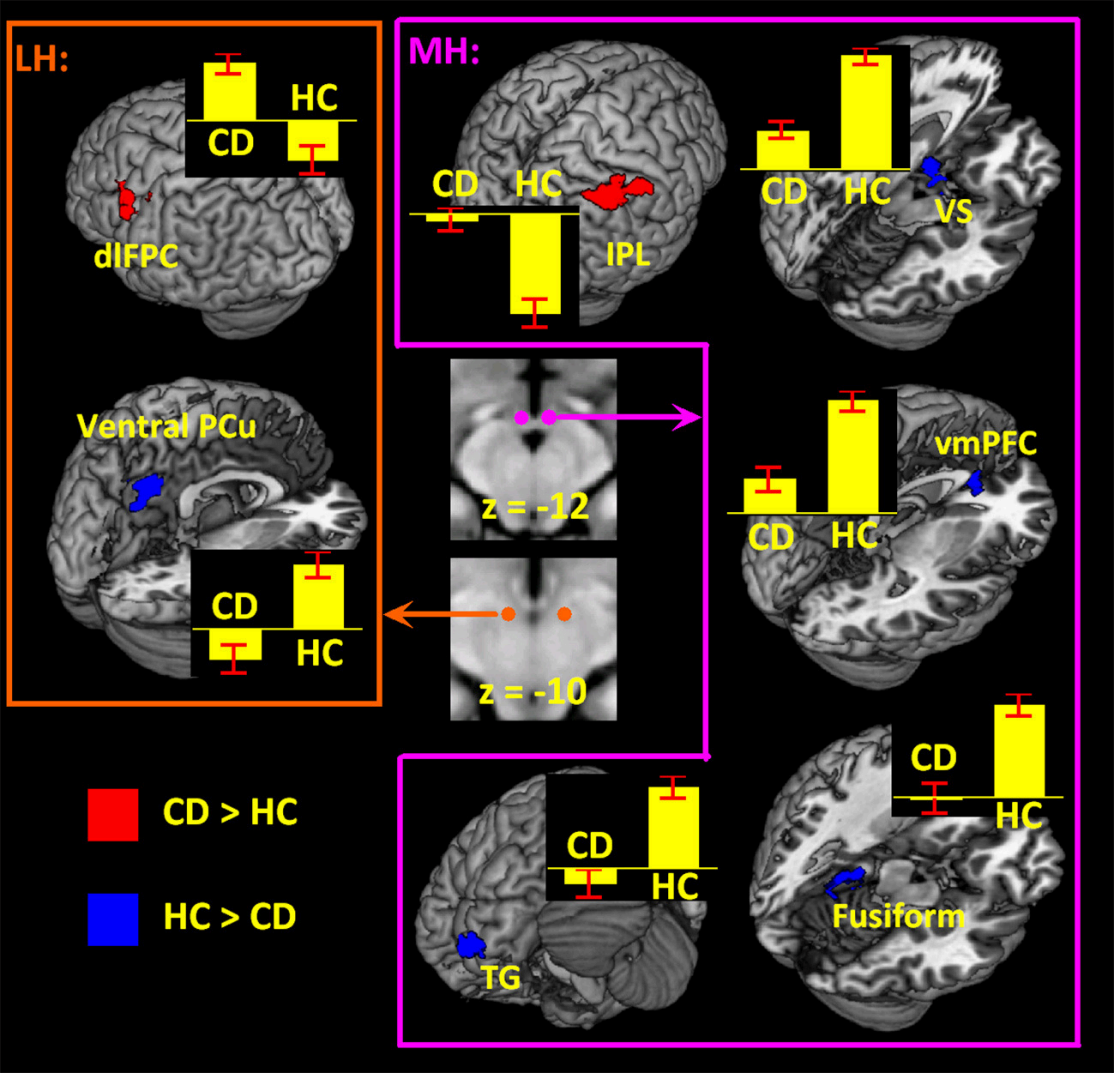
Hypothalamic connectivity differences between 70 CD and 70 HC (voxel *p* < 0.001 uncorrected and cluster *p* < 0.05 FWE corrected) each for LH and MH. Histograms show mean ± standard error of the connectivity *z* scores. dlPFC, dorsolateral prefrontal cortex; PCu, precuneus; IPL, inferior parietal lobule; VS, ventral striatum; vmPFC, ventromedial prefrontal cortex; TG, temporal gyrus.

Examined at voxel *p* < 0.05 corrected for FWE on the basis of Gaussian random field theory, the results of a one-sample *t*-test of the MH and LH connectivity to the whole brain are shown in Supplementary Figure 2 for HC and CD. The patterns of whole-brain connectivity largely reflected those reported earlier (66). We then compared CD and HC in a two-sample *t*-test at voxel *p* < 0.001 uncorrected and cluster *p* < 0.05 FWE (Figure 2; Table 2). Because more CD than HC participants were current smokers, we included smoker status as well as age as covariates in the model. Compared to HC, CD showed increased MH rsFC with inferior parietal lobule (IPL) as well as decreased rsFC with the ventromedial prefrontal cortex (vmPFC), ventral stratum (VS), superior temporal gyrus, fusiform gyrus and cerebellum. Compared to HC, CD showed increased LH rsFC with the dorsolateral prefrontal cortex (dlPFC) and decreased LH rsFC with the ventral precuneus (PCu).

**Table 2:** Regions showing differences in connectivity of MH and LH between 70 CD and 70 HC.

**Volume**	**Peak voxel**	**MNI coordinate (mm)**			**Side**	**Identified brain region**
**(mm**^3^**)**	**(Z)**	***x***	***y***	***z***		
***MH (CD > HC)***						
3,645	4.21	54	−31	46	R	Inferior parietal louble
***MH (HC > CD)***						
1,215	4.67	−21	−34	−17	L	Fusiform gyrus
	4.38	−24	−43	−20	L	Cerebellum
1,188	4.38	−48	14	−32	L	Temporal gyrus
1,080	4.02	6	35	−2	L/R	Ventromedial prefrontal cortex
1,107	4.15	−9	8	−14	L	Ventral striatum
***LH (CD > HC)***						
1,512	4.84	−48	11	31	L	Dorsolateral prefrontal cortex
***LH (HC > CD)***						
2,322	4.23	−3	−61	43	L/R	Ventral precuneus

*Voxel p < 0.001 uncorrected and cluster-level p < 0.05, FWE; R, right; L, left*.

**Figure 3:**
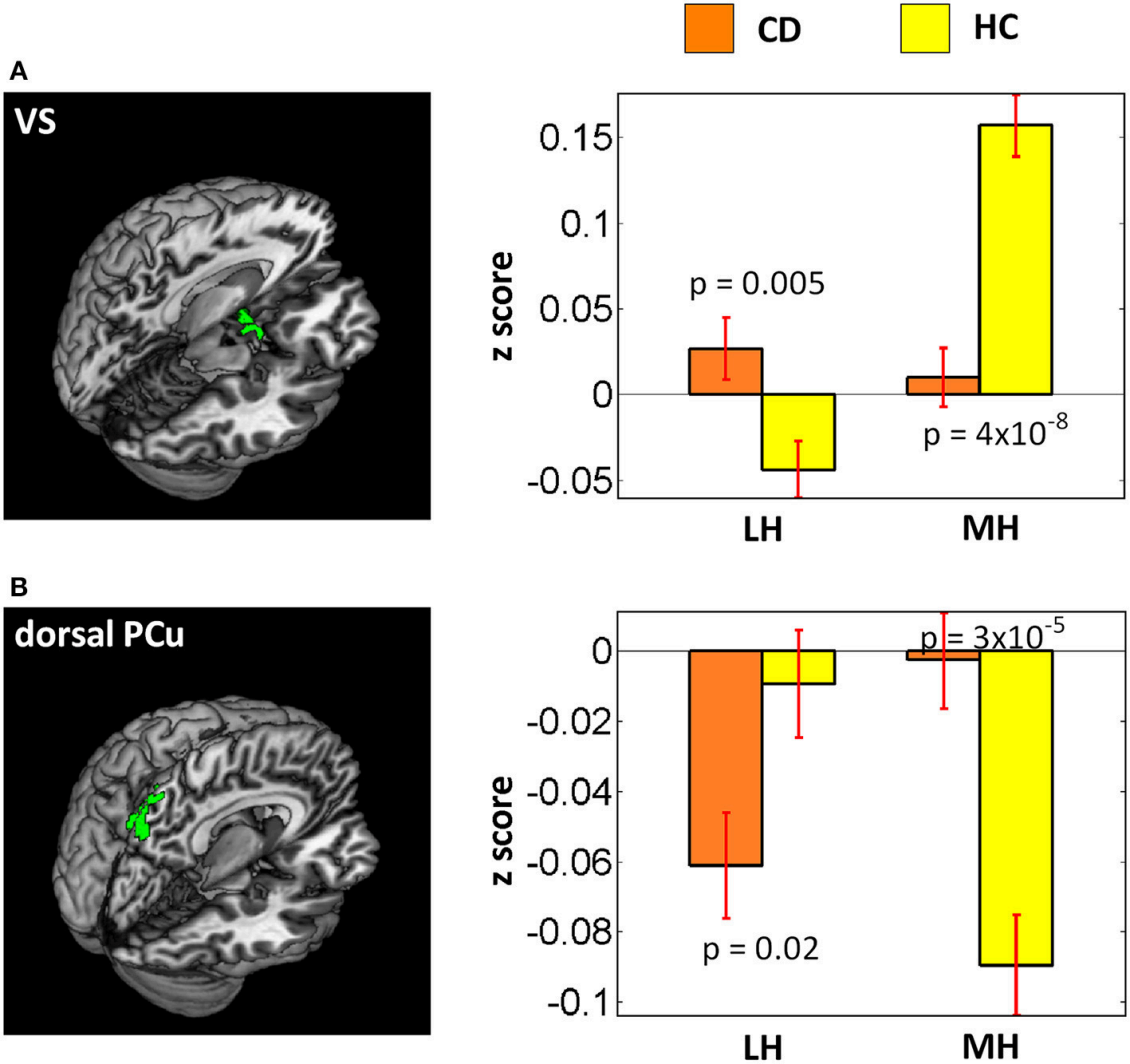
Results of two-way ANOVA (CD vs. HC × LH vs. MH). Left panel showed brain regions (**A**: ventral striatum; **B**: dorsal precuneus) identified under voxel *p* < 0.001 uncorrected and cluster-level *p* < 0.05 FWE corrected. Right panel showed bar plots of connectivity z scores (mean ± standard error). *P*-values were from planned two-sample *t*-tests of CD vs. HC each for LH and MH connectivity.

As the LH and MH appear to support motivated behavior in opposite directions, we conducted a two-way group (CD vs. HC) × seed (LH vs. MH) ANOVA with smoker status and age as covariates to examine the interaction effects. At voxel *p* < 0.001, uncorrected and cluster *p* < 0.05 FWE, the results showed main effects of increased dlPFC and decreased vmPFC, temporal gyrus, fusiform gyrus and cerebellum connectivities in CD as compared to HC, as expected. In interaction effects, the ventral striatum (VS, *x* = −9, *y* = 8, *z* = −17, volume = 891 mm^3^, peak voxel Z = 3.92) showed increased LH connectivity (*p* = 0.005) but decreased MH connectivity (*p* = 4 × 10^−8^) in CD as compared to HC (Figure 3A). In contrast, a cluster in the dorsal precuneus (PCu) (*x* = 6, *y* = −73, *z* = 49, volume = 999 mm^3^, peak voxel Z = 3.85) showed decreased LH connectivity (*p* = 0.02) but increased MH connectivity (*p* = 3 × 10^−5^) in CD as compared to HC (Figure 3B).

### Relationship to clinical characteristics

In evaluating the relationship between the connectivity changes and cocaine use variables, we focused on cocaine craving (CCQ score), duration of use (years), and amount of recent use (grams of cocaine used in the past month). Thus, with the number (7) of ROIs tested, the results of linear regression were evaluated with a corrected *p* = 0.05/(3 × 7) = 0.0024. None of the Pearson regressions demonstrated a correlation at the corrected threshold. At an uncorrected threshold, across CD, the LH-dlPFC connectivity strength (*z* scores) was positively correlated with total amount of cocaine use in the past month (*p* = 0.006, *r* = 0.34). The MH-VS connectivity strength was negatively correlated with cocaine craving as assessed by the CCQ (*p* = 0.006, *r* = −0.34). The MH-vmPFC connectivity strength was negatively correlated with years of cocaine use (*p* = 0.03, *r* = −0.26).

### Sex differences

**Figure 4:**
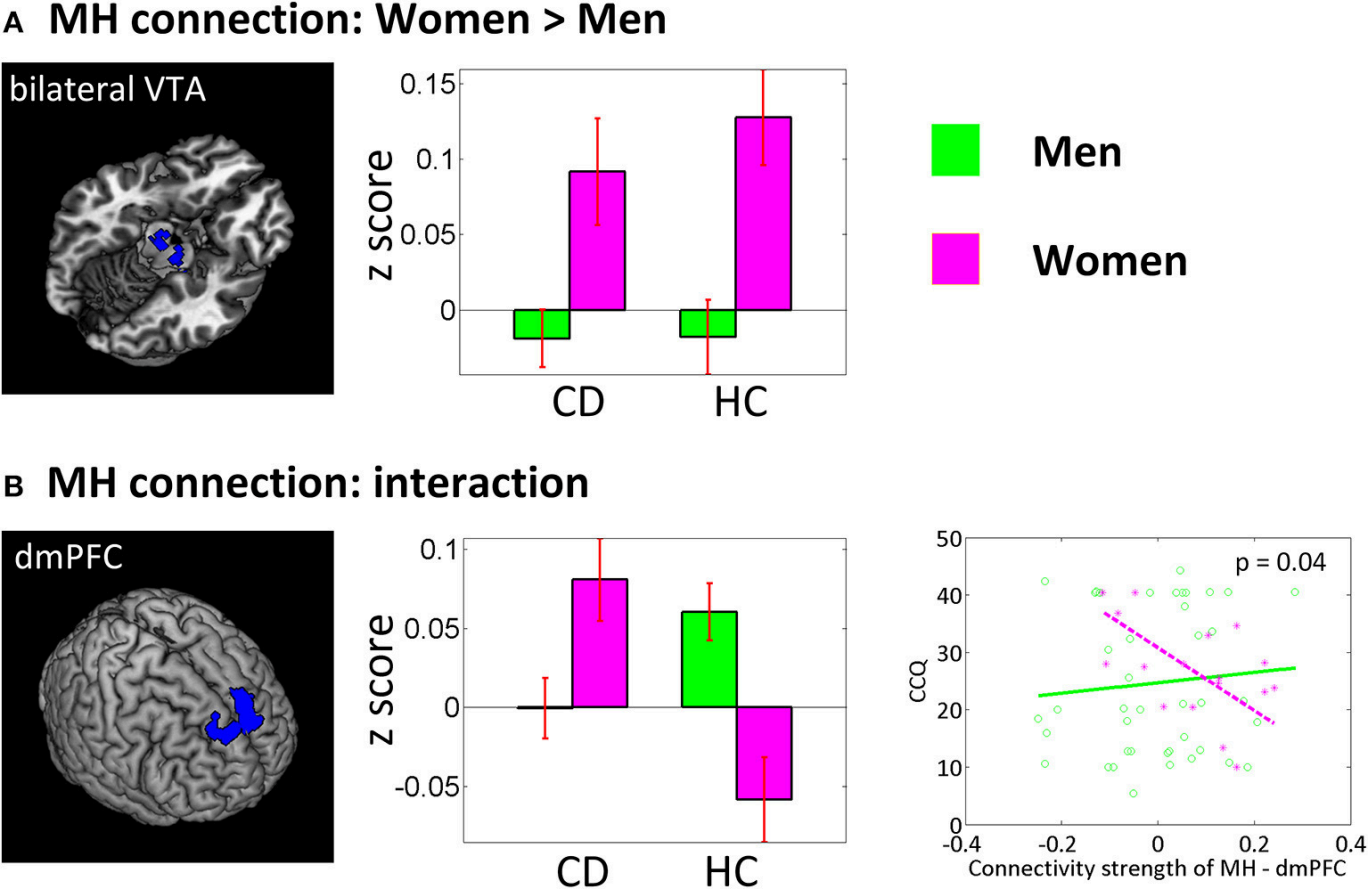
Sex main and sex by group interaction effects of a two-way ANOVA (CD vs. HC × men vs. women). Left panel showed brain regions identified under voxel *p* < 0.005 uncorrected and cluster-level *p* < 0.05 FWE corrected (or small volume correction at *p* < 0.05, FWE for the VTA). The middle panel showed corresponding bar plots of the connectivity strength (*z* score, mean ± S.E.). VTA, ventral tegmental area; dmPFC, dorsomedial prefrontal cortex. The lower right panel showed regression of CCQ score vs. MH-dmPFC rsFC for men vs. women.

In a separate ANOVA, we assessed group by sex interaction each for LH and MH connectivity with smoker status and age as covariates (Figure 4). For the sex main effect women showed higher connectivity between MH and bilateral ventral tegmental area (VTA) (*x* = 12, *y* = −19, *z* = −14, volume = 486 mm^3^, peak voxel Z = 3.73; and *x* = −9, *y* = −13, *z* = −17, volume = 540 mm^3^, Z = 3.35). An interaction effect was observed for the MH—dorsomedial prefrontal cortex (dmPFC) (*x* = −12, *y* = 38, *z* = 46, volume = 3,321 mm^3^, Z = 4.30) connectivity, with HC men showing higher connectivity than HC women and CD showing the opposite pattern. At an uncorrected threshold, MH-dmPFC connectivity was negatively correlated with CCQ score in CD women (*p* = 0.02, *r* = −0.64) but not in CD men (*p* = 0.59, *r* = 0.09). A slope test confirmed the sex difference (*p* = 0.04).

## Discussion

The ventral striatum is a major projection target of the dopaminergic midbrain and critically implicated in the etiology of cocaine addiction. Many studies have examined functional connectivities of the ventral striatum (98–102). We recently employed difference mapping to describe how ventral striatal subregions were altered in resting state connectivities in cocaine addicted vs. non-drug using individuals (103). Hypothalamus also receives heavy dopaminergic projections from the midbrain. On the other hand, no studies to date have examined whether or how functional connectivities of the hypothalamus may be influenced by chronic cocaine exposure. The current work took on this challenge and explored resting state functional connectivity (rsFC) of the lateral (LH) and medial (MH) hypothalamus in recently abstinent individuals with cocaine dependence (CD) as contrasted with age- and sex-matched healthy subjects (HC). Whole-brain rsFC of the LH and MH largely replicated the results reported earlier, with the LH connected with the midbrain, thalamus, lentiform nucleus as well as the rostral and dorsal anterior cingulate cortex and the MH connected with the ventral striatum and ventromedial prefrontal crotex (66). Compared with HC, CD showed distinct changes in LH and MH rsFC. We highlighted some of the main findings in the below.

### Hypothalamus and ventral stratum (VS)

The VS was more heavily connected with the MH than LH, in accord with (66). Compared to HC, CD showed decreased MH-VS connectivity, with the connectivity strength negatively correlated with cocaine craving score. Thus, cocaine addiction is associated with diminished MH-VS connectivity and the extent of impairment appears to be associated with cocaine craving. The VS and hypothalamus are reciprocally connected (104). Both the VS and hypothalamus receive projections from the dopaminergic midbrain and are implicated in processing of reward-related stimuli, including those associated with drugs of abuse (6, 14, 104–106). Imaging studies showed that the extracellular concentration of dopamine in the VS increased with administration of drugs of abuse, and the experienced euphoria was positively associated with the extent of dopamine release in the VS (107–110). Hypothalamus connectivity with the VS was also observed here as a group (CD vs. HC) by seed (LH vs. MH) interaction effect, with CD showing increased LH and decreased MH-VS connectivity as compared to HC. For HC, the VS was each positively and negatively connected with the MH and LH, but this differential pattern of connectivity was not observed in CD. How disrupted hypothalamus VS connectivity may conduce to changes in motivated behavior in CD would be an important issue to address in future work.

### Hypothalamus and precuneus

Compared with HC, CD showed decreased LH rsFC with the ventral precuneus. ANOVA showed an interaction effect with CD and HC each showing stronger negative LH and MH connectivity with the dorsal precuneus. Previous studies implicated the precuneus in behavioral engagement and self-awareness, with the ventral precuneus comprising part of the default mode network (DMN) (111–114). DMN dysfunction has been widely reported in drug addiction (115–120) and hypothalamus—DMN dysconnectivity was observed in a number of neuropsychiatric disorders (121–124). The current findings may also be considered with our earlier report of the fractional amplitude of the low frequency BOLD signal of the dorsal precuenus as a neural analog of behavioral engagement (113) and studies of altered behavioral engagement in cocaine misuse (125–129).

### Hypothalamus and prefrontal cortex (PFC)

The hypothalamus and nucleus accumbens share projections from the PFC (130), with the medial PFC projecting preferentially to the MH (131). Neurons of the subgenual anterior cingulate cortex—a subarea of the vmPFC—increased firing during rest and sleep (132), as do neurons of the MH (133, 134). Neurons in the vmPFC that project directly to the hypothalamus responded to a conditioned food cue in sated rats (135). Both the vmPFC and hypothalamus responded to emotional exposure (136), sexual arousal (42, 44, 45, 137), as well as food stimulus (138, 139). These studies suggested functional coordination between the (medial) hypothalamus and vmPFC, which, as the current findings showed, may be disrupted in CD.

The dlPFC is widely implicated in drug addiction. Severity of drug use across heroin, alcohol, MDMA, and cannabis was negatively associated with dlPFC activation in dependent individuals (140). In the current study, we observed increased functional connectivity between the LH and dlPFC, a finding that can be considered with earlier reports of hyperactive dlPFC during negative emotion processing in addicted individuals (29, 128, 141–144). In studies of depression, repetitive transcranial magnetic stimulation of the dlPFC induced neuroendocrine changes, likely via its effects on the hypothalamus-pituitary-adrenal (HPA) axis (145). Interestingly, a recent study showed increased hypothalamic connectivity with the dlPFC in depression patients after 8 weeks of sertraline treatment (146). Thus, altered LH-dlPFC connectivity may reflect an outcome of chronic exposure to catecholaminergic agents, including cocaine and many antidepressants. How cocaine misuse disrupts dlPFC regulation of the HPA axis activity warrants more research.

### Hypothalamus and inferior parietal lobule (IPL)

We observed increased MH rsFC with the IPL in CD as compared to HC, with HC but not CD showing significant negative connectivity. The finding was broadly consistent with attention and parietal dysfunction in chronic cocaine users (147–151). Notably, a recent study showed increased hypothalamus IPL connectivity during exposure to food vs. non-food stimuli in obese individuals (152). Further, craving and hypothalamus—IPL connectivity both decreased after leptin administration, in support of a functional role of this circuit in food “wanting.” MH-IPL circuit dysfunction may similarly be investigated with cue craving tasks or behavioral paradigms that address attentional bias to drug cues.

## Limitations of the study, conclusions, and future research

Several limitations of the study need to be considered. First, the hypothalamus is a relatively small structure (~2,360 mm^3^, vs. amygdala ~3,744 mm^3^, for comparison). We drew a hypothalamus mask in MNI space based on a brain atlas (65) and marked the putative locations of hypothalamic subnuclei based on Baroncini et al. (20), as shown in Supplementary Figure 1. Our LH and MH seeds were within the range of previous reported coordinates. The connectivity we observed between LH and dmPFC/thalamus and between MH and vmPFC/ventral striatum was identical to the results reported in Kullmann et al. (66). On the other hand, image resolution as well as inter-subject variation in anatomy pose challenges to accurately identifying the hypothalamus. These findings thus remain to be substantiated. Second, CD may differ from HC in details of alcohol and nicotine use, or other clinical variables, such as history of childhood trauma, that were not assessed in the current study. Further, although candidates with clinical depression or anxiety disorders were excluded, we evaluated subclinical depression and anxiety with the BDI and STAI for CD but not for HC. Thus, we cannot rule out the possibility that the current findings may be influenced by these other clinical variables. Third, the 10-min resting state scan was conducted after four 10-min sessions of a stop signal task to address cognitive control. These task runs may influence rsFC of the default network, for instance (153). Although a previous study also showed that whole brain functional connectivity organizations were robust and stable between pre- and post-task resting states (154), we cannot rule out the possibility that the hypothalamus rsFC may be influenced by prior task runs. Fourth, as described earlier, the hypothalamus is involved in a variety of motivated behavior. Here, we related the connectivity findings to cocaine use variables but it remains to be seen how hypothalamus connectivity is disrupted in association with changes in of motivated behavior in cocaine addiction.

In summary, we demonstrated altered hypothalamus rsFC in recently abstinent cocaine addicted individuals. Cocaine misuse was associated with distinct changes in LH and MH rsFC. The findings suggested the importance in characterizing the role of hypothalamus circuit as a neural marker of cocaine addiction. Both thalamus and hypothalamus functions are disrupted in cocaine addiction (72, 155–159). Although studies have generally conceived of thalamus and hypothalamus dysfunction in terms of the respective effects of cocaine on noradrenergic and dopaminergic signaling, more work is needed to examine the neurobiological bases of thalamus-hypothalamus dysfunction in cocaine addiction.

## Author contributions

SZha and CL contributed to the conceptualization and design of the study. SZha, WW, SZho, and CL carried out the experiment and data analyses. All authors contributed to the writing and approved the final version of the manuscript.

### Conflict of interest statement

The authors declare that the research was conducted in the absence of any commercial or financial relationships that could be construed as a potential conflict of interest.
