# Supplementary material for: Resting State Functional Connectivity of the Lateral and Medial Hypothalamus in Cocaine Dependence: An Exploratory Study
Source: Front Psychiatry. 2018 Jul 27;9:344. doi: 10.3389/fpsyt.2018.00344 (PMC6072838; doi:10.3389/fpsyt.2018.00344)
Supplement: Supplementary file 1 [file Data_Sheet_1.pdf]

SUPPLEMENTARY MATERIALS

Zhang et al.:

Resting state functional connectivity of the lateral and medial hypothalamus in cocaine dependence: an exploratory study

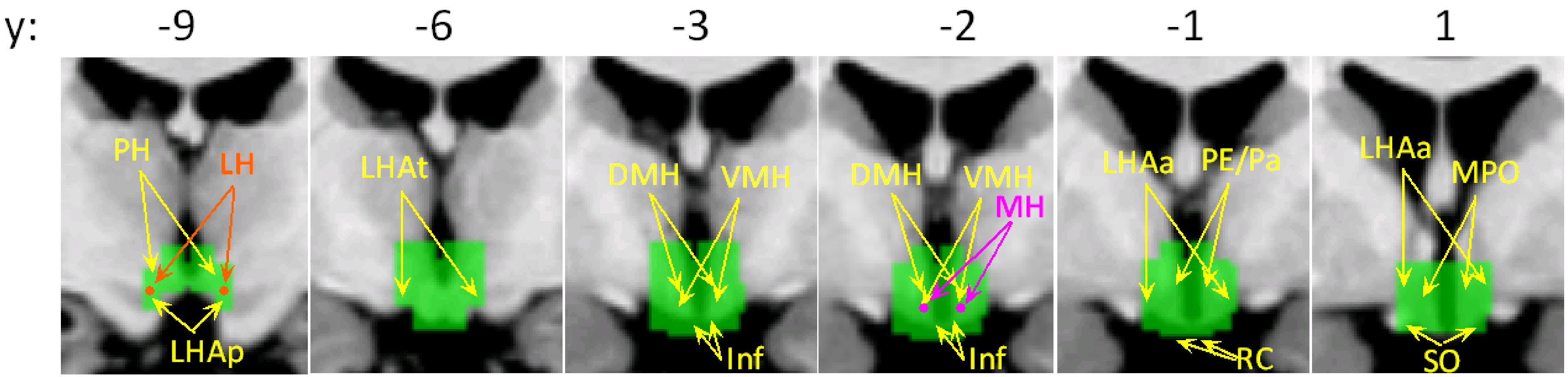

Supplementary Figure 1: Hypothalamic mask (2,360 mm^3) as well as marked subnuclei in MNI space according to Dr. Elanor Hinton (personal communication) and an atlas (Mai et al., 2007). LH: lateral hypothalamus seed; MH: medial hypothalamus seed; DMH: dorsomedial nucleus; VMH: ventromedial nucleus; LHAa, LHAp, LHAt: lateral hypothalamus (anterior, posterior, or tuberal part); PH: posterior hypothalamus in mammillary region; Inf: infundibular nucleus (arcuate nucleus); PE/Pa: periventricular/paraventricular nucleus; RC: retrochiasmatic nucleus; SO: supraoptic nucleus; MPO: medial preoptic nucleus. The LH and MH seeds were each of L and R spheres 2mm in radius and thus occupied 4 voxels (108 mm^3).

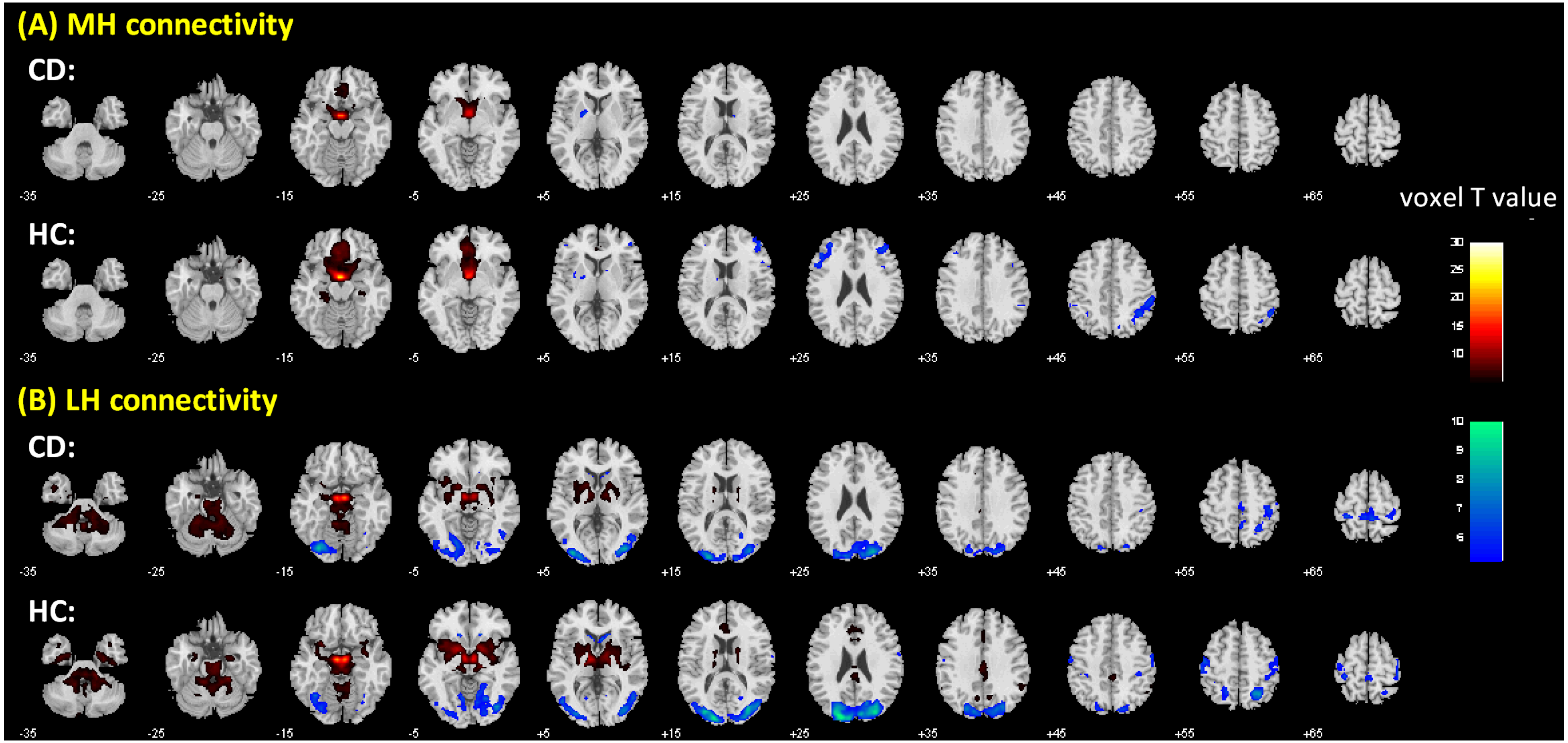

Supplementary Figure 2: Whole brain functional connectivity of the medial (A) and lateral (B) hypothalamus across 70 CD and 70 HC, one-sample t test, $p < 0.05$, FWE corrected. Warm color: positive correlation; cool color: negative correlation. The color bar represents voxel T values. The patterns of whole-brain connectivity largely reflect those reported earlier (Kullmann et al., 2014).

Kullmann, S., Heni, M., Linder, K., Zipfel, S., Haring, H.U., Veit, R., Fritsche, A., and Preissl, H. (2014). Resting-state functional connectivity of the human hypothalamus. *Hum Brain Mapp* 35**,** 6088-6096.
